# ROS Cocktails as an Adjuvant for Personalized Antitumor Vaccination?

**DOI:** 10.3390/vaccines9050527

**Published:** 2021-05-19

**Authors:** Ramona Clemen, Sander Bekeschus

**Affiliations:** ZIK, Leibniz Institute for Plasma Science and Technology (INP), Felix Hausdorff Str. 2, 17489 Greifswald, Germany; ramona.clemen@inp-greifswald.de

**Keywords:** antigen, cold physical plasma, gas plasma technology, immunogenicity, oxidative post-translational modifications, oxPTM, reactive nitrogen species, reactive oxygen species

## Abstract

Cancer is the second leading cause of death worldwide. Today, the critical role of the immune system in tumor control is undisputed. Checkpoint antibody immunotherapy augments existing antitumor T cell activity with durable clinical responses in many tumor entities. Despite the presence of tumor-associated antigens and neoantigens, many patients have an insufficient repertoires of antitumor T cells. Autologous tumor vaccinations aim at alleviating this defect, but clinical success is modest. Loading tumor material into autologous dendritic cells followed by their laboratory expansion and therapeutic vaccination is promising, both conceptually and clinically. However, this process is laborious, time-consuming, costly, and hence less likely to solve the global cancer crisis. Therefore, it is proposed to re-focus on personalized anticancer vaccinations to enhance the immunogenicity of autologous therapeutic tumor vaccines. Recent work re-established the idea of using the alarming agents of the immune system, oxidative modifications, as an intrinsic adjuvant to broaden the antitumor T cell receptor repertoire in cancer patients. The key novelty is the use of gas plasma, a multi-reactive oxygen and nitrogen species-generating technology, for diversifying oxidative protein modifications in a, so far, unparalleled manner. This significant innovation has been successfully used in proof-of-concept studies and awaits broader recognition and implementation to explore its chances and limitations of providing affordable personalized anticancer vaccines in the future. Such multidisciplinary advance is timely, as the current COVID-19 crisis is inexorably reflecting the utmost importance of innovative and effective vaccinations in modern times.

## 1. Introduction

Each year 14.1 million new cases of cancers are diagnosed that require therapeutic attention. The classic pillars in oncology are surgery, radiotherapy, and chemotherapy. These measures have markedly improved median survival in patients across all types of cancer. However, significant progress has slowed down in the past decades for several reasons, radioresistance and chemoresistance being among them [1,2]. Meanwhile, biologicals, such as cytokines and antibodies targeting growth receptors, spurred therapy success [3,4,5]. A paradigm shift in oncology then came with the incorporation of antitumor immune defense into the treatment concepts and repertoires of the field of oncology. Although being predicted in the 1960s already [6,7], the concept needed several decades, and a leap in life science technology innovations, along with mechanistic concepts in immunology and oncology to harness its full potential. Today, antibodies targeting immunosuppressive checkpoint receptors on T cells have provided substantial clinical responses [8]. Their success, along with the Nobel Prize Award in Physiology and Medicine in 2018 for achievements in this field, has given antitumor T cells undisputed importance across the globe for providing tumor protection [9]. Tumor protection is carried out by generating antigen-specific T cells, followed by strengthening one’s immune response through a specific anti-tumor immune response. The personalized antigen vaccines have recently come to the fore [10] because they can be efficient and have few side effects. However, there are novel ways to optimize therapeutic anti-tumor vaccines in various strategies, such as a modified tumor biopsy vaccine [11], cryptic peptide [12], nano-particle loaded [13], or a PEG-modified antigen vaccine. Here we propose a new technical approach to optimize the immunization by mimicking a relevant biological process of the inflammatory microenvironment, namely the generation of reactive species.

## 2. Tumor Immune Evasion and Vaccination

Cancers evolve under the constant pressure of the immune system; a process called immune evasion [14]. Tumor variants with minimal activation of immune cells have a growth advantage over clonotypes with highly immunogenic antigens. This classic view was complemented over the last two decades with the opposite scenario. Highly immunogenic tumor cells do not attempt to hide from immune recognition but counteract immune cell activation by activating immunosuppressive ligands and receptors, for example, PD-L1, PD-L2, and CD80/86 [15]. Other mechanisms of an immunosuppressive microenvironment complement this camouflage and sabotage. For instance, hypoxia [16], soluble mediators such as kynurenine [17], and the promotion of suppressive immune cell subsets including M2 macrophages and regulator T cells [18]. However, the clinical success of checkpoint antibodies targeting receptors and ligands suggests the receptor-ligand-based immunosuppression of effector T cells as being a critical determinant of the therapeutic outcome. Hence, it is clear that strengthening the activity of *existing* antitumor T cell clones is a proven therapeutic concept in cancer immunotherapy.

A second complementary approach is broadening the T cell receptor repertoire by augmenting the generation of *novel* antitumor T cell clones. Autologous tumor vaccines provide a vast array of tumor-associated antigens (TAA) and neoantigens to the host. Such antigens are present in all types of tumors, albeit to a varying degree [19]. Another limitation is that not all of these tumor antigens are presentable on major histocompatibility complex (MHC) molecules due to the preference of protein digestion and peptide cleavage in the proteasome and immunoproteasome [20,21], as well as the affinity of the MHC receptor family towards specific amino acids of the peptide to provide suitable binding affinity [22,23]. However, the critical determinant of generating T cell activation or tolerance towards such antigens is the inflammatory context in which these are presented, along with the efficacy of antigen presentation. To address these issues, dendritic cells (DCs), being professional antigen-presenting cells, have been investigated in numerous preclinical and clinical studies for their ability to promote antitumor immunity after being loaded with tumor antigens in vitro [24,25]. Undoubtedly, this elegant type of cell therapy fostered the understanding of tumor immunology in oncology and benefited many patients enrolled in clinical trials. Nevertheless, this concept also has limitations. First, there was limited success in many clinical trials. Second, DC loading and expansion require state-of-the-art facilities and are associated with high costs. Even if near-ideal protocols had been, or were to be, developed, it is still questionable whether DC therapy would become a global gold standard for cancer therapy apart from in countries with privileged income and health care systems. Third, much focus has been put on DC activation and maturation. Simultaneously, the conditioning of the tumor material has received less attention, as was recently well demonstrated in a cohort of cancer patients [26], which at least gives rise to the idea of rethinking the inevitable need of DCs in the realm of tumor vaccination.

Textbook immunology predicts that the body has an inherent interest in mounting both B cell and T cell immune responses against (non-self) antigens if presented in a sufficiently inflammatory context. Adjuvants provide the latter, being the basis of vaccinations, a process currently receiving significant interest duringthe COVID-19 pandemic. Together, with the points mentioned above, this raises the question of what is limiting the use of autologous tumor material to be directly used as a vaccine without the need for external processing by other cell types. It is understood that early and recent attempts of using a native autologous tumor vaccination to provide therapeutic efficacy [27,28] failed. Notwithstanding, we here outline why reactive oxygen and nitrogen species might be a fascinating option to render tumor antigens more suitable for direct vaccination campaigns in oncology and possibly adjuvant to existing strategies [29,30,31,32], which are numerous and not covered here. It should be stressed that, in the tumor context, this text always refers to therapeutic vaccinations and not preventive/prophylactic vaccination.

## 3. Reactive Oxygen and Nitrogen Species

Reactive oxygen and nitrogen species (ROS/RNS) are molecules with great reactivity and abbreviated with ROS in this work, as most RNS contain oxygen. Besides their past underappreciation as mere metabolic byproducts, ROS are pivotal intracellular redox signaling agents [33], critical for infection control [34], and increasingly recognized as key elements of the inflammatory microenvironment. Immune cell activation and metabolic reprogramming of leukocyte subsets have been linked to endogenous ROS production as crucial to driving these processes [35,36,37,38]. Perhaps the best-known role of non-constitutive ROS is their early appearance during inflammation by immune cells and non-immune cells alike. ROS release is the very first event during tissue damage [39] and it is required for the subsequent neutrophil influx. Subsequent neutrophil priming and activation auto-amplifies ROS production, followed by another round of ROS amplification by incoming monocytes and macrophages that complement the reactive species array with several nitrogen species [40].

For instance, nitric oxide synthase (NOS) produces nitric oxide (NO), which reacts with superoxide (O_2_^−^), that is generated by NADPH (nicotinamide adenine dinucleotide phosphate) oxidases (NOX), to yield peroxynitrite (^-^ONOO). The enzyme superoxide dismutase (SOD) catalyzes the reaction of superoxide to hydrogen peroxide (H_2_O_2_). In the presence of hydrogen peroxide, the arterial indoleamine 2,3-dioxygenase 1 (IDO-1) formates singlet oxygen (^1^O_2_) for blood pressure regulation and vascular tone during inflammation [41]. In the presence of iron, H_2_O_2_ promotes the generation of highly reactive hydroxyl radicals (HO^.^) in the Fenton reaction [42]. Furthermore, myeloperoxidase (MPO) is known to generate hypobromous acid, hypochlorous acid, and hypothiocyanite. The hypochlorite radicals can participate in the formation of atomic oxygen (O) and HO [34]. This is the environment in which infection-related antigens are recognized, modified, and transported to the secondary lymphatic organs to activate adaptive immunity.

Current vaccine preparation strategies almost unanimously neglect this ancient evolutionary part of antigen modification. When taking a view into other research fields, this comes as a surprise. For decades, researchers have identified a pivotal role of ROS and oxidative post-translational modifications (oxPTMs) in autoimmunity [43]. Chronic inflammation and chronic ROS release modified antigens, leading to auto-antibodies and auto-reactive T cells that are observed in numerous diseases, including rheumatoid arthritis, systemic lupus erythematosus, and diabetes [44,45,46,47,48], partly in a neoepitope-like fashion [49,50]. Mechanistically, oxPTMs have been ascribed a function similar to damage-associated molecular patterns (DAMPs) [51], providing pro-inflammatory stimuli in in professional antigen-presenting cells (APCs), and are decisive for the balance between antigen tolerance and immunity. Altogether, multiple ROS modify antigens, leading to a DAMP-like character to activate innate immunity and potentially neoepitopes to broaden adaptive immunity and the B cell and T cell receptor repertoire. ROS are, therefore, ideal candidates to increase the immunogenicity of autologous tumor vaccines. However, the challenges of working with ROS are numerous. First, their production, reaction kinetics, and specificity are hard to control, apart from the short half-lives associated with most species. Second, oxidative modifications are challenging to track and require sophisticated infrastructure and bioinformatics for their analysis. Third, and most notably, a simultaneous generation of several highly reactive compounds is technically impossible unless utilizing a concept from physics: gas plasma technology.

## 4. Gas Plasma Technology as a Significant Innovation in Generating Multi-ROS Cocktails

**Figure 1:**
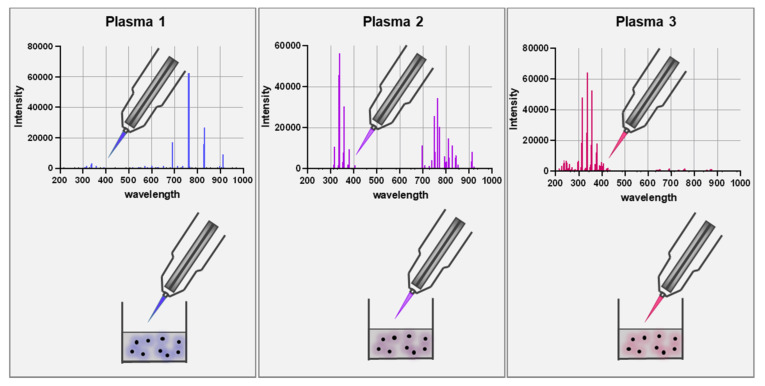
Scheme of using different feed gas settings to generate gas plasma with distinct ROS cocktail profiles. The upper panel represents optical emission spectroscopy (intensity: relative units; wavelength: nanometer) measurements of the visible plasma effluent leaving the jet device. The lower panel is a schematic of a biological target being exposed to the gas plasma resulting in distinct oxidative modification patterns as depicted with the color code.

Gas plasma is an electron-impact and photon-driven technology. In gas plasma jets, usually, a noble gas is excited by a high-frequency electrode [52]. Excited noble gas species transfer their chemical energy to oxygen and nitrogen in the ambient air, generating vast amounts of several reactive oxygen and nitrogen species simultaneously. Compared to hot gas plasma, cold plasmas are operated at body temperatures and therefore do not denature proteins or harm cells and tissue by thermal energy transfer. Therefore, the main product is the bio-active multi-ROS cocktail [53,54,55]. Similar to the ROS released during inflammation, plasmas generate short-lived species (O, •NO, •NO, O_2_•^-^, •OOH, ^-^ONOO, ^1^O_2_, etc.) as well as long-lived molecules that are mostly deterioration products from short-lived species such as H_2_O_2_, NO_2_^−^, NO_3_^−^, and HOCl [56,57]. Hundreds of chemical reactions have been identified in gas plasma jets using computer modeling, and redox biology currently does not offer the tools to identify each of the reaction products unambiguously. The degree of complexity is increased when considering the different spatio-temporal concentrations of each of the species along the axis of a plasma jet. Nevertheless, gas plasmas are unique in their ability to deliver multi-ROS cocktails onto biologically relevant targets. Strikingly, the ROS cocktail can be modified by changing the gas composition fed into the plasma jets (Figure 1). This leads to an enrichment of some types of ROS and a partial depletion of others [58,59]. This way, unique oxidative modification patterns are being generated at biological target molecules, as recently shown for the model peptide cysteine using mass spectrometry [60]. Additionally, prototypic plasma jets often allow other parameters to be tuned, for instance, the feed gas flux, the excitation frequency and wave form, and the input power. Other studies confirmed the modification of antigens and proteins by plasmas [61,62], leading to functional changes [63,64,65].

## 5. Proof-of-Concept Study Using Multi-ROS Cocktails to Provide Vaccine Tumor Control

In a recent study, we used chicken ovalbumin (Ova) to study the immunogenicity of multi-ROS cocktails in vitro and in vivo [66]. Using transgenic OT-II mice harboring Ova-specific T cells, we found gas plasma modified Ova to elicit significantly enhanced T cell activation compared to native antigen (Ova). This effect was specific, as it could not be replicated using human albumin. Splenocytes of other mice strains also did not show any elevated T cell activation. Strikingly, the enhanced T cell activation seen with gas plasma-treated Ova was not recapitulated when modifying Ova with equimolar amounts of long-lived reaction products from the plasma in treated liquids (H_2_O_2_, NO_2_^−^, NO_3_^−^, HOCl), unambiguously pointing towards a role of the unique cocktails generated by short-lived species. One plasma condition had more substantial effects than another one, which—in this specific setting—suggested a role of singlet and atomic oxygen or, possibly, lower ozone levels in providing immunogenic oxidative modifications. Using mass spectrometry, dozen of different modifications (e.g., oxidation, dioxidation, trioxidation, chlorination, and quinones) were found at many of the over 400 amino acids. This exemplifies the high degree of complexity, especially when considering the multi-ROS nature of the gas plasma system, currently making it difficult to come to a specific conclusion on which modifications have what effect. Some modifications were also in the sequence of the cognate peptide region. Moreover, it is possible (and likely, in our hands) that our observations were not based on one single type of modification or amino acid, but were rather a result of several modifications, complicating the control and understanding of this tool, as of now. We also found that oxidatively modified full Ova protein was needed, as the treatment of the oxidated immunogenic peptide alone (27 amino acids) did not elevate T cell responses. All this notwithstanding, the in vivo findings clearly showed functional consequences of the multi-ROS exposure to the antigen. In naïve mice, an increased anti-Ova T cell activity was created when using oxidized over native Ova, which was also reflected in a more inflammatory cytokine release profile. Notably, the gas plasma-derived multi-ROS Ova antigen oxidative modification led to significantly decreased tumor growth of Ova-expressing melanoma cells when given as a vaccine in a prime-boost scheme, compared to native untreated Ova. This was accompanied by the higher numbers and activation profiles of intratumoral T cells. These results emphasize the power of the multi-ROS antigen modification concept.

## 6. Concept and Challenges of Multi-ROS-Modified Autologous Tumor Vaccines

We propose gas plasma technology to upgrade antitumor vaccines by increasing adjuvanticity and antigenicity: the former due to the DAMP character of antigen oxPTMs promoting DC activation, as in the concept of immunogenic cell death (ICD) [67,68]. Indeed, gas plasma technology was shown to induce ICD [69], change proteomics suggesting neoepitope presentation [70], and increase the activity of antigen-presenting cells [71]. The increased immunogenicity of oxPTM and the potential formation of neoantigens was observed in autoimmunity [47,48]. However, the development of autoimmune disorders is not always based on oxidized antigens. Instead, some antigens are native [72], citrullinated [73,74] or deaminated [75]. Interestingly, Hultqvist and colleagues have shown that elevating the low oxidative burst capacity led to suppressing an autoimmune response [76,77]. With gas plasma technology, we mimic the ROS production of an oxidative burst to modify tumor-associated antigens.

**Figure 2:**
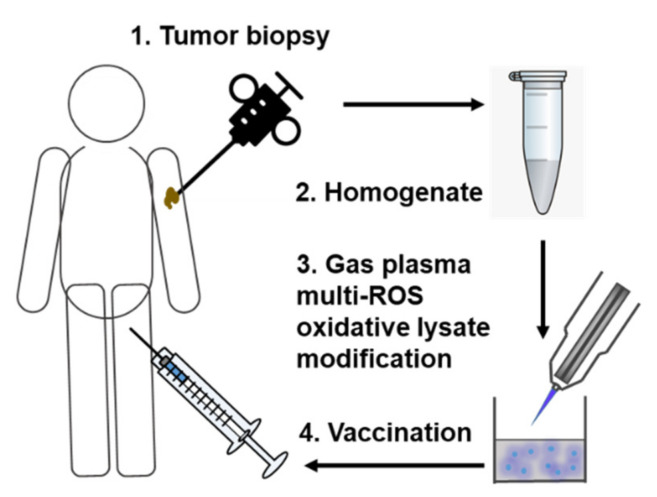
Simplified scheme of gas plasma technology-mediated multi-ROS-driven improvement of autologous tumor vaccines. After tumor biopsy and homogenization of the tumor material, oxidation with complex multi-ROS cocktails generated by medical gas plasma jet technology follows prior to vaccination.

We propose to homogenize collected autologous tumor tissue, followed by gas plasma exposure of the tumor lysates in a defined and pre-optimized setting (Figure 2).

The lysates may be stored frozen in several aliquots until used in multiple vaccination rounds. Next, the multi-ROS oxidized homogenates can be thawed and combined with a pre-optimized adjuvant; a process that could be performed in a quality-controlled environment, such as pharmacies. In a series of elegant studies, the team of Lana Kandalaft employed HOCl-oxidized whole tumor lysates fed to autologous DCs, followed by the therapeutic vaccination of the latter, in a cohort of ovarian cancer patients [26]. Hundreds of vaccines generated in this way were well-tolerated without serious side effects. This study, which has been preceded by a decade of research [78,79,80,81,82,83,84,85,86], is the clinical proof-of-concept that oxidation of autologous tumor antigen potentiates antitumor immunity. Since, in our setting, optimized gas plasma exposure was even enhanced compared to the effect of HOCl, an additional benefit of multi-ROS modification might be feasible. So far, there have been three studies using gas plasma-inactivated tumor cells as a preventive/prophylactic vaccine that significantly decreased the tumor growth of live tumor cells given 7–9 days after vaccination [87,88,89]. Elevated tumor-infiltrated T cells with memory phenotypes were shown in plasma-treated tumors, and in patients, the infiltrated immune cells correlated with a better outcome [90,91]. As mechanisms of action, ICD was also recently proposed to improve DC antitumor vaccinations [92,93]. Further motivating our approach, there have been promising results with oxidized mannan-MUC1 (Mucin 1, cell surface associated; CD227) vaccination, as concluded from a 15-year follow-up study showing significantly fewer recurrences [94].

It is understood that there are many degrees of freedom in this concept. The ideal ROS cocktail needs to be identified. The gas plasma technology exposure needs to be implemented in a quality-managed environment. Either the necessity of DCs or the ideal adjuvant needs to be established, along with questions on optimized absolute dosing, injection frequency, administration routes, and potential deterioration of the ROS-treated vaccine. There might also be interdependence between these parameters. For instance, it was recently reported that a sequential intravenous priming vaccination followed by a later intradermal boost vaccination showed the best effects in providing antitumor immunity in mice, while simultaneous administration significantly worsened the outcome [95]. Apart from these practical aspects, the scientific challenges lie in the mapping of the type and number of the oxidative antigen modifications, the identification of optimal ROS cocktails for maximizing immunogenicity, the elucidation of putative APC receptors needed to recognize oxidized antigen, and the clarification of the role of the proteasomal and antigen-presenting machinery activity to stimulate cognate antigen recognition optimally in the host. The T cell activation and differentiation are dependent on the binding affinity between epitope and MHC-molecules and between MHC-complex and T cell receptors [96,97]. Consequently, the presentation of oxidatively modified antigens can alter binding affinities [74], possibly resulting in an increase or even decrease in T cell activation. After all, even a presumably perfect antitumor vaccine cannot circumvent tumor microenvironments that are hostile or suppressive to T cells. Therefore, vaccination should be embedded in a treatment strategy that also addresses these challenges. Finally, augmenting antitumor immunity, especially when using whole tumor lysates containing mostly self-antigens, is always at the verge of promoting autoimmunity. However, at least in the trials performed by Kandalaft and colleagues, such adverse events were not observed [81].

## 7. Conclusions

Therapeutic autologous antitumor vaccination is an elegant way of providing personalized therapy in oncology. However, its efficacy and practicability are limited by different constraints, and new enhanced vaccine technologies are required. Due to the current lack of validated biomarkers and neoantigens, upgrading a biopsy of the tumor is an intelligent, time-saving, and cost-effective way to enable personalized therapy. Oxidizing and modifying autologous tumor material with multiple reactive oxygen and nitrogen species simultaneously seems a promising avenue to increase both antigenicity (enhanced T cell receptor repertoire) and immunogenicity (increased co-stimulation and activation of adaptive antitumor immunity). Medical gas plasma jet technology is a recent innovation capable of providing multi-ROS cocktails in a unique and equivocal manner. Here, it is proposed to consider implementing this novel tool and to advocate its potential and limitations in providing efficient, fast, and affordable autologous antitumor vaccination, not just to those in privileged health care systems.

## Data Availability

Not applicable.
